# Roles of the semisolid heating on the microstructure of a hypereutectic Al–Fe–Cu alloy

**DOI:** 10.1038/s41598-021-80983-5

**Published:** 2021-01-15

**Authors:** Wei Wang, Bo Liu

**Affiliations:** School of Marine Engineering Equipment, Zhejiang Ocean University, Zhoushan, 316022 People’s Republic of China

**Keywords:** Engineering, Materials science

## Abstract

A hypereutectic Al–Fe–Cu alloy with a high-volume fraction ferro-aluminum second phase (AlFe phases for short) was reheated in the solid–liquid region and the microstructure evolution was investigated. During semisolid heating, the high-melting AlFe phases in the Al–Fe–Cu alloy were demonstrated to stunt the grain growth and to block the liquid coalescing and the solid moving. Consequently, the grain sizes in the alloy increased rapidly and then slowly with increasing holding time, and the grains increased gradually with increasing temperature. Smaller grain grew into the large grain but it did not continually grow into the larger grain with increasing temperature or holding time. The shape factor (***SF***) of the alloy increased gradually and then decreased quickly with increasing temperature or holding time. The major alloying elements in addition to magnesium in the hypereutectic Al–Fe–Cu alloy were finally enriched at the grain boundaries or around the AlFe phases. Besides dissolving in the grains or AlFe phases, copper also diffused between the grains or around AlFe phases, resulting in the formation of diverse Cu-enriched zones. Cu constituents in the inter-grains are outnumbered in the intra-grains. The coarsening kinetics of the alloy was controlled by grain boundary diffusion. The coarsening rate constants ***K*** in the initial stage of heating (5–20 min) were several times larger than that in the later stage of heating (20–60 min), indicating the blocking effect of AlFe phases on coarsened grain being obvious.

## Introduction

Al–Fe alloys are superior lightweight materials that are used in a wide range of applications where excellent heat resistance, corrosion resistance, wear resistance, and high strength-to-weight ratios are desirable and are fully recyclable such as transportation and aerospace industries. Furthermore, the maximum solubility of iron in aluminum is 0.05 wt% at the eutectic temperature of 655 °C (an equilibrium binary Al–Fe system) and is even lower at room temperature. Most of the iron in Al alloys is present in the form of lamellate or acicular fragile intermetallic compounds. The properties of a specific alloy depend on the physical, chemical, and stereological properties of its main phases and precipitates, that is, phases with Fe and precipitates. Therefore, reducing the negative effect of phases with Fe and improving comprehensives of Al–Fe alloys have become a key factor in determining its potential for wide adoption.

The semisolid forming of the hypereutectic Al–Fe–Cu based alloys could alter the morphology of phases with Fe and decrease the negative effect of the phases, making they be appropriate for the stressed parts such as the bracket, the connecting rod, the slider, etc., working at the temperature of 500 K or so^1–3^. The semisolid forming technique is an effective approach to inexpensively manufacture alloys containing a high-volume fraction second phase.

Each step of semisolid processing has a great effect on the following processes^4^: the coarsening of migrating grain boundary liquid films in which the coarsening rate is relevant to the solid volume fraction, where the coarsening rate decreases as the solid fraction increases before reaching a critical value and then decreases over this critical value^5,6^, coalescence for alloys of high solid fraction and aggregation for grains with low disorientation boundaries or to reduce the free energy, is primarily a lattice diffusion-controlled process^7–10^. For many diffusion-controlled coarsening systems, including solid–liquid mixtures, coarsening kinetics can be described by the Lifshitz, Slyozov and Wagner (**LSW**) theory as follows:

1 $$ D^{n} - D_{0}^{n} = Kt $$

where ***t*** is the isothermal holding time, ***D*** is the grain size after time* t*, ***D***_**0**_ is the initial grain size, ***K*** is the coarsening rate constant, and ***n*** is the power exponent^11^. In general, ***n*** is 2, 3, and 4 representing an interfacial reaction-controlled coarsening, a volume diffusion-controlled coarsening, and a grain boundary diffusion-controlled coarsening, respectively^12^. The variation of alloy microstructure heated in the liquid–solid region appeared to be related to the initial microstructure, grain morphology, structure of grain boundaries, alloy composition, etc.^4,13^.

Semisolid alloys produced by different routes have various initial microstructures. The coarsening rate constant, ***K***, value increases as the isothermal treatment temperature increases. However, the* K* values for spray-cast alloys, ECAPed (Equal Channel Angular Pressed) alloys and the FSPed (Friction Stir Processed) alloys, which there were higher misorientation angles between adjacent grains in the initial microstructure of these alloys, are less than those for alloys produced by strain induced melt activated (SIMA) or recrystallization and partial melting (RAP)^4,13^, indicating that the initial structure in alloys strongly affects the coarsening behavior in the liquid–solid region. Furthermore, alloys with similar grain sizes and different grain boundary structures, such as Spray-forming 7034 Al alloy^14^ and SIMA 7075 Al alloy^15^, have different coarsening rate constants, and Spray-forming 7034 Al alloy with more HAGBs (High Angle Grain Boundary) have much lower ***K*** values than those of the SIMA 7075 Al alloy in heating the liquid–solid region. There is clearly a significant effect of the grain boundary structure on grain coarsening in the solid–liquid region. Alloys with higher misorientation angles between grains can indirectly restrain the grain coarsening of semisolid alloys^4^.

Moreover, the isolated, non-wetting, and larger granules as well as the thermally stable dispersoid in alloys, the dispersoid size is larger than the thickness of the grain boundary liquid films, would either drag along or pin to migrating grain boundary liquid films or inhibit the liquid diffusing from one boundary position to another. The insoluble particles or phases with Fe, Mn, and Si in 7075, 319, and/or 2014 Al alloys as well as nano-sized SiC particles in 7075 alloy were proven to be more effective for barrier migration of the liquid or to pin to grain boundaries than those of the soluble Al_2_Cu particles in alloys^7,13,15–17^, resulting in a decrease in the coarsening rate when heating the solid–liquid region.

In addition, during semisolid heating, the meltage first occurred in the eutectic zone on the boundaries between the initial grains and then penetrated the polygon boundaries, leading to the separation of the initial grains into small new grains^18–20^. Subsequently, the grains were transformed into globular or near-globular shapes under the action of the solid–liquid interfacial tension^8,19,20^, and evolved or agglomerated into larger and irregular shapes with increasing holding time or heating temperature. The α-Al grains were quickly coarsened by connecting the secondary arms of the rosette or merging fine α-Al phases with a small quantity of the liquid, and slowly coarsened through diffusion at the stage of a large quantity of the liquid^9,10^. Some reinforced precipitations that are rich in copper, magnesium, and zinc in the 7075 alloy^21^, or rich in copper in the Al–5Fe–4Cu alloy^22^ are low-melting intermetallics. Additionally, these are distributed at grain boundaries and/or within grains, forming the intergranular liquid resulting in the intergranular gathering of strengthening elements in semisolid heating, which would significantly influence the results of subsequent solution treatment of Al–5Fe–4Cu alloy^22^.

Al–Fe semisolid alloys have nondendritic microstructures consisting of globular or subspherical α-Al solid, liquid matrix and numerous infusible lamellate and blocky ferro-aluminum phases with or without other elements, which are collectively referred to as AlFe phases and which their cusp could obtund, and the longer phases were dehisced into several gaps as a result of dissolving during heating in the liquid–solid region^2,23^, and prepared for subsequent thixoforming, which can strongly crush and thin the solid, especially on the brittle AlFe phases, in the semisolid slurry^24,25^. However, there are a few studies on the microstructure evolution of semisolid heating for this type of Al alloy. Understanding the behavior of hypereutectic Al–Fe–Cu alloys in solid–liquid regions is more complicated than that of conventional semisolid aluminum alloys. The micro-behavior of the alloy in the solid–liquid region has a significant impact on their properties, accordingly, it is necessary to understand and recognize this behavior. In this work, a hypereutectic Al–Fe–Cu alloy was produced by electromagnetic stirring first, and then the microstructure evolution was investigated in the semi-solid temperature range in detail. Finally, the effect of AlFe phases in the alloys on the grain coarsening as well as the segregation of primary alloying elements in the semi-solid state were also discussed.

## Results and discussion

### Effect of heating in the solid–liquid region on grain size

**Figure 1:**
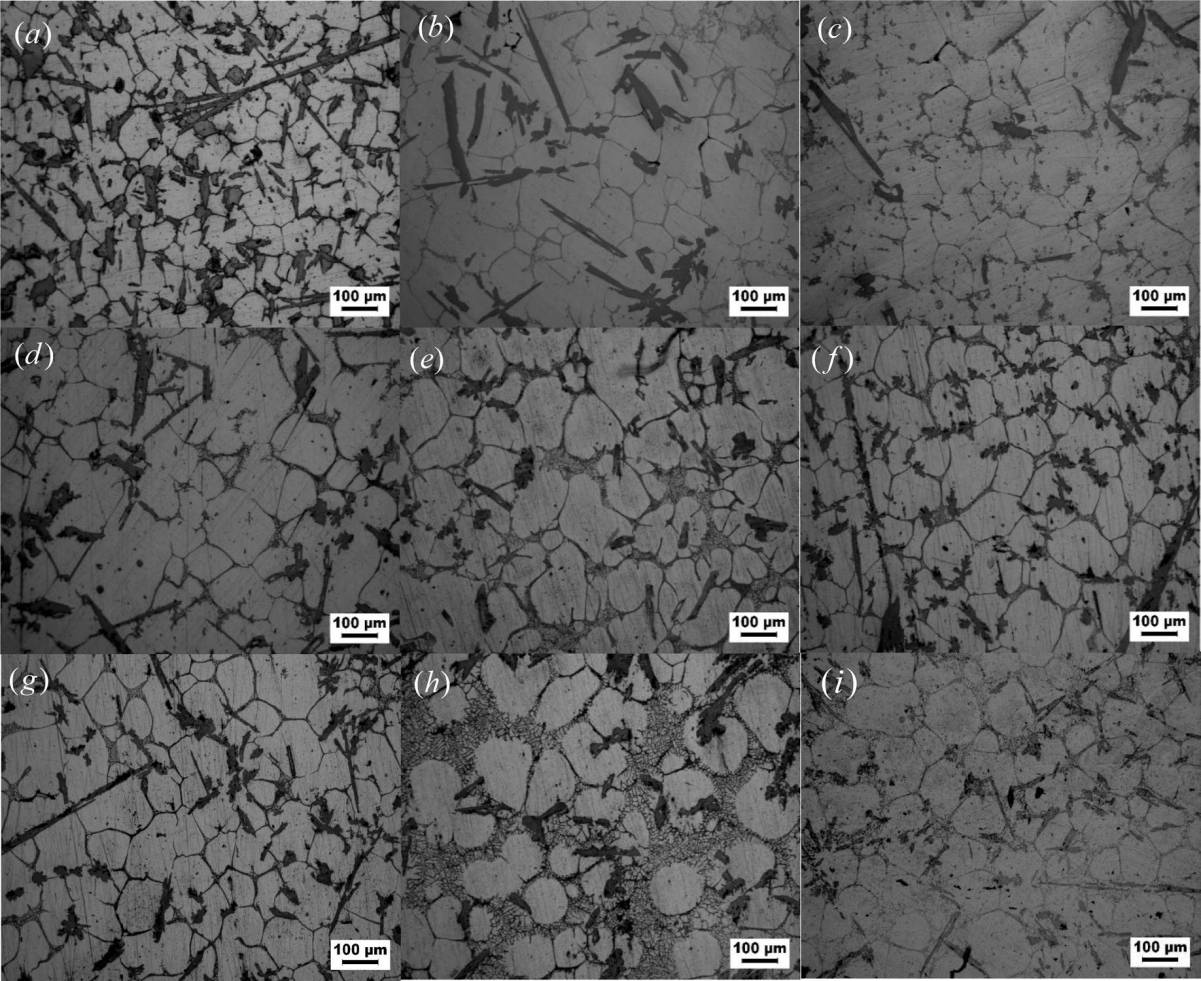
Microstructure of Al–Fe–Cu alloy quenched water after semisolid heating for different temperatures and times. (**a**) At 620 °C for 5 min, (**b**) at 630 °C for 5 min, (**c**) at 640 °C for 5 min, (**d**) at 620 °C for 30 min, (**e**) at 630 °C for 30 min, (**f**) at 640 °C for 30 min, (**g**) at 620 °C for 60 min, (**h**) at 630 °C for 60 min, (**i**) at 640 °C for 60 min.

**Figure 2:**
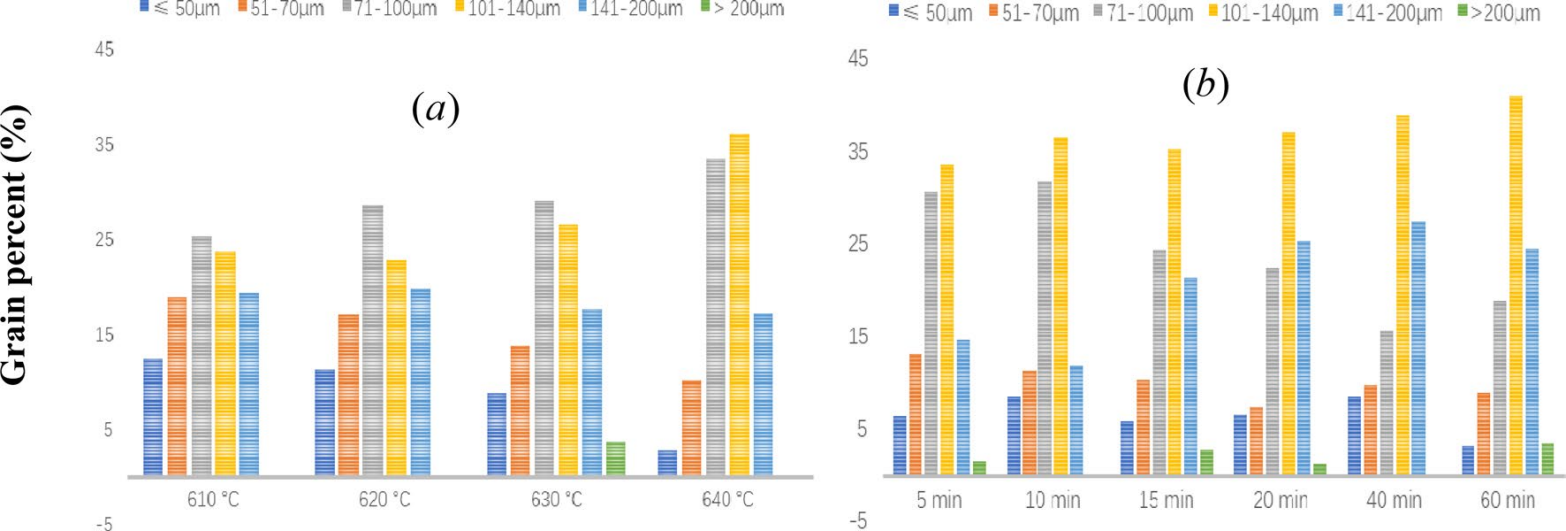
The percent composition of the grains in Al–Fe–Cu alloy held for 30 min at different temperature (**a**), and heated at 630 °C for various holding times (**b**).

Figure 1 describes the water quenched structure of the Al–Fe–Cu based alloy after heating in the solid–liquid region at different temperatures and times. The grain sizes in the alloy varied from 50 to 200 μm, the number of grains over 200 μm was low, and that under 50 μm was fewer during the semisolid heating. The metallographic observation revealed that the grain sizes from 70 to 140 μm predominated in the Al–Fe–Cu alloy. The smallest grains (< 50 μm) diminished rapidly, the largest grains (> 200 μm) did not vary significantly, and the grains increased gradually with increasing heating temperature. The smallest grains (< 50 μm) were almost unchanged, and the largest grains (> 200 μm) had slightly increased as the holding time increased at the same temperature (Fig. 2). The smaller grains grew into large grains, but it did not continue to grow into larger grains. The average grain size enlarged quickly and then increased slowly at each temperature with increasing holding time, indicating that the solid AlFe phases in the Al–Fe–Cu alloy would impede or restrain the grains developing farther.

A deviation of grain distribution between Fig. 2a,b may be related to the constituent and microstructure of specimens, the distribution density of AlFe phases in a field of view, the viewing fields chosen, the grains selected in fields of view, and so on. This does indicate that the distribution of the remolten liquid zones in the alloy is not uniform due to the presence of AlFe phases, resulting in that grains are not uniform in size or shape. However, the trend of the grain size increasing with increasing heating temperature or holding time is invariant.

### Evolution of the shape factor and the equivalent diameter in the semisolid heating

**Figure 3:**
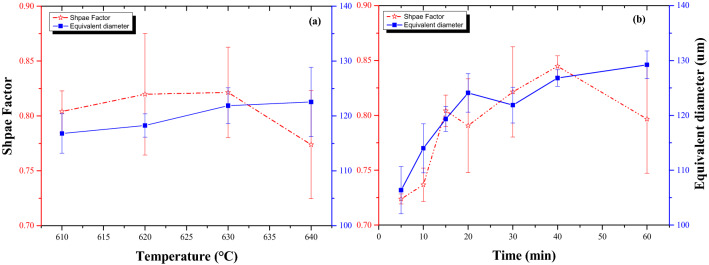
Shape factor (SF) and the equivalent diameter of the grains of Al–Fe–Cu based alloy with various temperatures and times. The imaginary line and the solid line represent Shape factor (SF) and Equivalent diameter, respectively. (**a**) Holding on 30 min; (**b**) heating at 630 °C.

Figure 3 shows the change in the shape factor (***SF***) and the average grain size (***D*** equivalent diameter) as a function of the heating temperature and holding time. With increasing the heating temperature or holding time, the shape of the solid particles becomes more globular, but the ***SF*** decreases quickly after reaching a maximum value. The ***SF*** and the average grain size can indirectly evaluate the thixotropy of the semisolid slurry, because ***SF*** strongly influences the flow ability and viscosity of the material. Generally, opportune lengthening the holding time or elevating the temperature in the liquid–solid region conduced to rounding the grains further, that is, the circularity of the grain increases with increasing heating temperature and holding time. The ***SF*** of the solids increased from 0.80 to 0.82 as the isothermal temperature increased from 610 to 630 °C, indicating that the grains became more orbicular. On samples heated at 630 °C, the ***SF*** increased with increasing holding time, and their shapes rounded further. At 40 min, the ***SF*** reached a maximum value, which showed the best globularity in the semisolid slurry. Subsequently, the ***SF*** decreased significantly with increasing holding time, resulting in the formation of larger grains with irregular shapes and deteriorating the circularity of the solid grains. Furthermore, the grains rounded gradually and the grains in the as-reheated alloy were more orbicular than those in the as-electromagnetic-stirred alloy^2^, but the grain size did not develop further and the equivalent diameter increased slightly with increasing heating temperature. Upon heating at 630 °C, the grains grew rapidly in the initial stage (up to 20 min) followed by slight increase with lengthening holding time. There was a clear inflection point, indicating that the grain coarsening was quick and then slow. Additionally, the circularity of the grains would deteriorate with increasing heating temperature for longer holding times.

The study indicated that the optimum processing parameters for Al–Fe–Cu alloy during reheating in the solid–liquid region are heating between 625 and 635 °C and holding 20–40 min, fitting with following thixoforming.

### Effect of the AlFe second phase on grain growth

Al–Fe–Cu alloy contains a high-volume fraction of the infusible AlFe phases. These AlFe phases are mainly elongated and flaky and are distributed randomly throughout the matrix. Some of the longest AlFe phases may be several times longer than the α-Al grains, but there are fewer in the as-electromagnetic-stirred alloy. The partial melting or dissolution of the cusps and the sides of the AlFe phases, as well as the local fusing of some longer AlFe phases during heating in the solid–liquid region resulted in the AlFe phase in the as-reheated alloy being more diminutive than that in the as-electromagnetic stirred alloy^2,24^. The average length of the AlFe phases was comparable to the grain size in the Al–Fe–Cu alloy, but the proportion of AlFe phases over 200 μm was higher than that of grains, accounting for more than 10%^26^.

**Figure 4:**
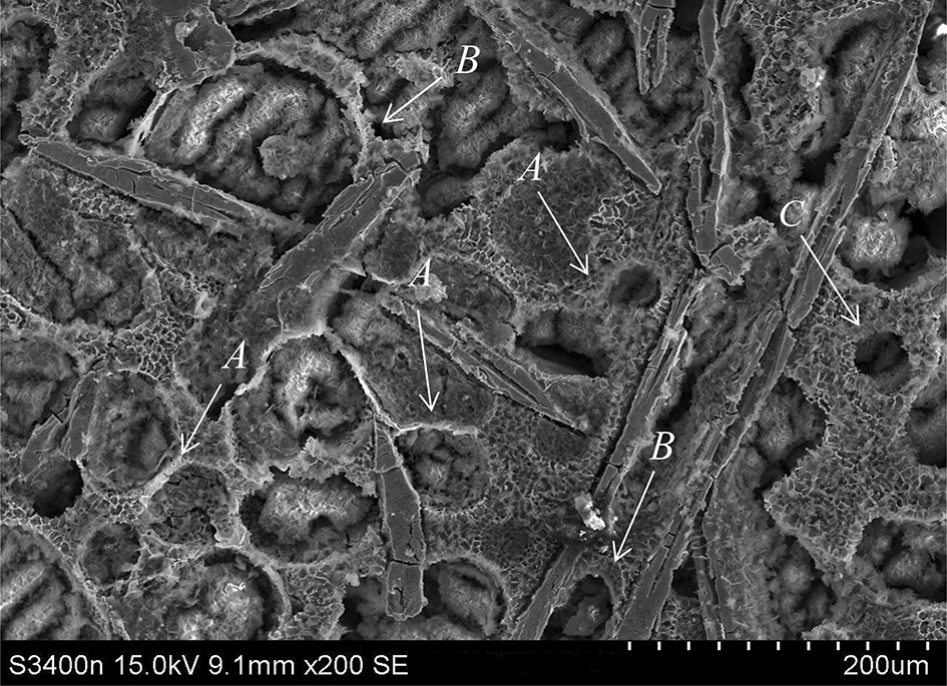
Microstructure of Al–Fe–Cu alloy was quenched after heating at 630 °C for 40 min, deep etched. The α-Al grain was corroded into a cavity with a white rich-Al brim, the re-solidified region presented the fine mesh structure. A, B and C micro-zones have various distributed condition of the liquid pools.

During heating in the solid–liquid region, the Al–Fe–Cu alloy’s matrix was randomly divided into many the miniature zones by these insoluble AlFe phases. These zones are roughly classified into three categories depending on the distribution of the liquid around the grain caused by the various distribution density of the AlFe phases. As shown in Fig. 4, in the most of the conditions that the AlFe phases distribution presented relatively uniform, Type A, there was much liquid around the solid particles, and the liquid filled the pools between the grains but partial interconnection between the liquid pools. There was a little the liquid able to diffuse from one pool to another, resulting in heterogeneous constituents in the pool. The granular brim near the AlFe phase would grow along the lamellate phases, making the grains develop unequally (see A in Fig. 4). In a small quantity of the conditions that the AlFe phases distribution presented dense, Type B, there was a slight liquid near the solid that was not fully wrapped by the liquid. The grains grew towards the liquid. The granular brim between AlFe phases would finish growing after meeting the lamellate phases, and the brim steeped in the liquid would develop into an arc (see B in Fig. 4). A grain’s interface located in rich liquid was rounding and that in poor liquid became noncircular, so that the granular shape became irregular. In other conditions that the AlFe phases distribution presented sparse, Type C, the grains were completely surrounded by the liquid pools that were interconnected, and the liquid was distributed evenly around the grains. The liquid could migrate sufficiently between these pools, resulting in a relatively homogeneous constituent in the zones. The grains developed uniformly in size and in shape, observably improving the circularity of the grains. Moreover, the grains were smaller and more symmetrical because of the dual obstruction of the liquid and AlFe phases (see C in Fig. 4). These smaller grains could only grow to a certain degree with increasing heating temperature or soaking time, indicating that the quantity of the largest grains in the alloy did not change obviously or had slightly increased with increasing heating temperature or holding time.

The inhibiting effect of AlFe phases in Al–Fe–Cu alloy on the grain coarsening resulted in a very small coarsening rate constant during reheating in the liquid–solid region. And, the inhibition of AlFe phases is not varying throughout heating process unlike that the restraint of Al_2_Cu or silicon particles disappeared as heating temperature increased or soaking time lengthened because of precipitating and dissolving^13,18^. During the grains growing or coarsening, if the grain boundary migrating encounters a barrier (as AlFe intermetallic), thus, the grain coarsening slackened or even ceased. Consequently, during heating in the liquid–solid region, the lamellate AlFe phases would hinder the grains coarsening and restrict the liquid diffusing, streaming and gathering, leading to the grains and the constituent unevenly.

### $B$Alloy element distribution in the solid–liquid region

**Figure 5:**
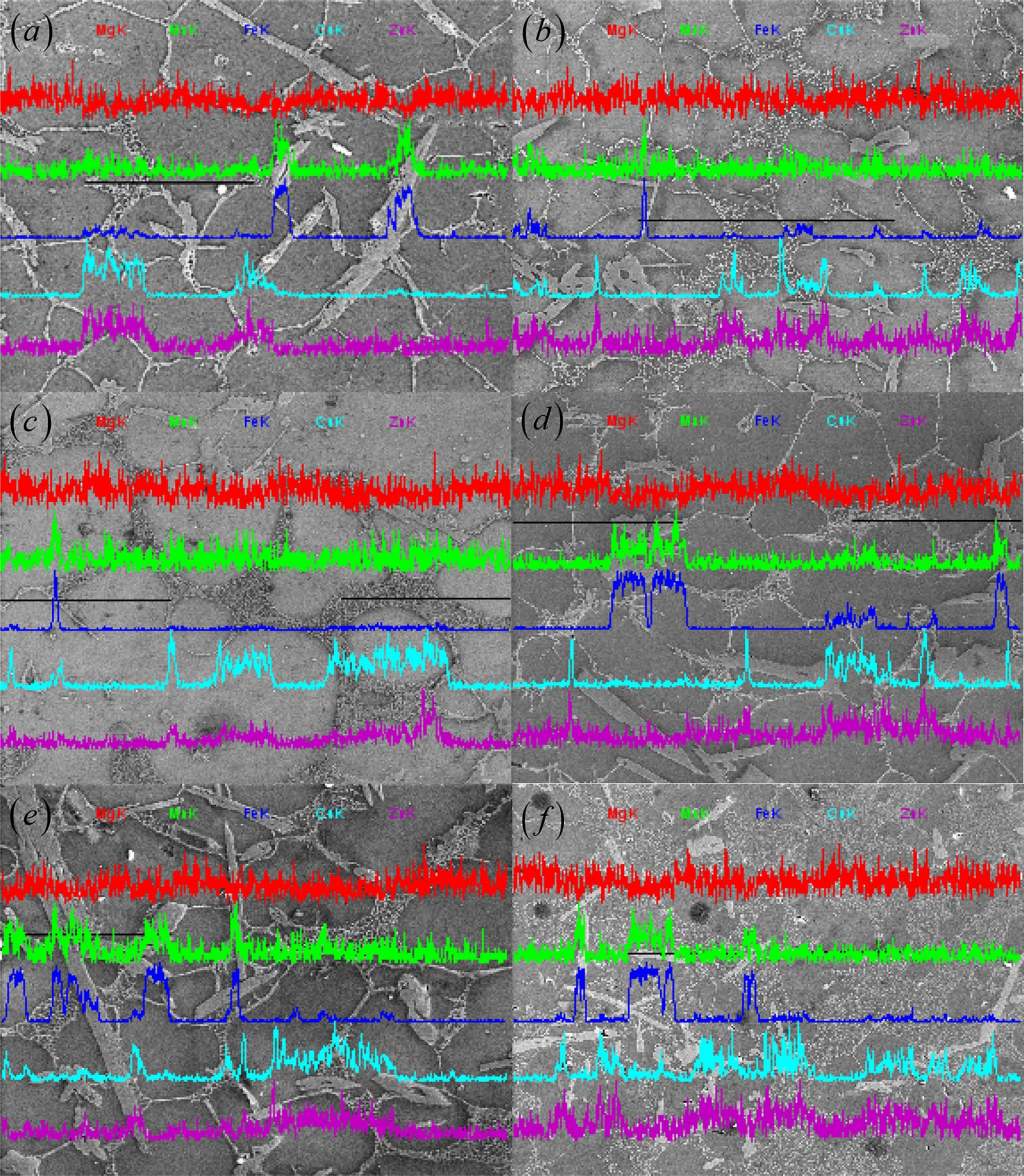
EDS analysis of Al–Fe–Cu alloy with isothermally held for 30 min at 640 °C (**a**), 630 °C (**b**), and 610 °C (**c**), and at 630 °C for 5 min (**d**), 15 min (**e**), and 60 min (**f**).

The Al–Fe–Cu alloy consisted mainly of iron, copper, zinc, manganese and magnesium elements. Iron only formed an insoluble iron-rich intermetallic (Al_13_Fe_4_ with or without Mn and Cu) with aluminum. The presence of the AlFe intermetallic would increase the resistance of the grain boundary movement and grain rotation, and could inhibit the liquid film migration during the liquid–solid region heating^15^. Furthermore, AlFe phases formed a dam-like structure due to the elongated and flaky morphology and then blocked the free flow of the low-melting liquid, resulting in isolated liquid pools in the interphase regions^16^. Figure 5 shows SEM micrographs and chemical composition line scans of the alloying elements in the Al–Fe–Cu alloy after remelting at different heating temperatures and soaking times. The EDS analysis revealed the increased levels of copper, zinc, iron, and manganese elements at the grain boundaries and/or around AlFe phases besides magnesium.

**Figure 6:**
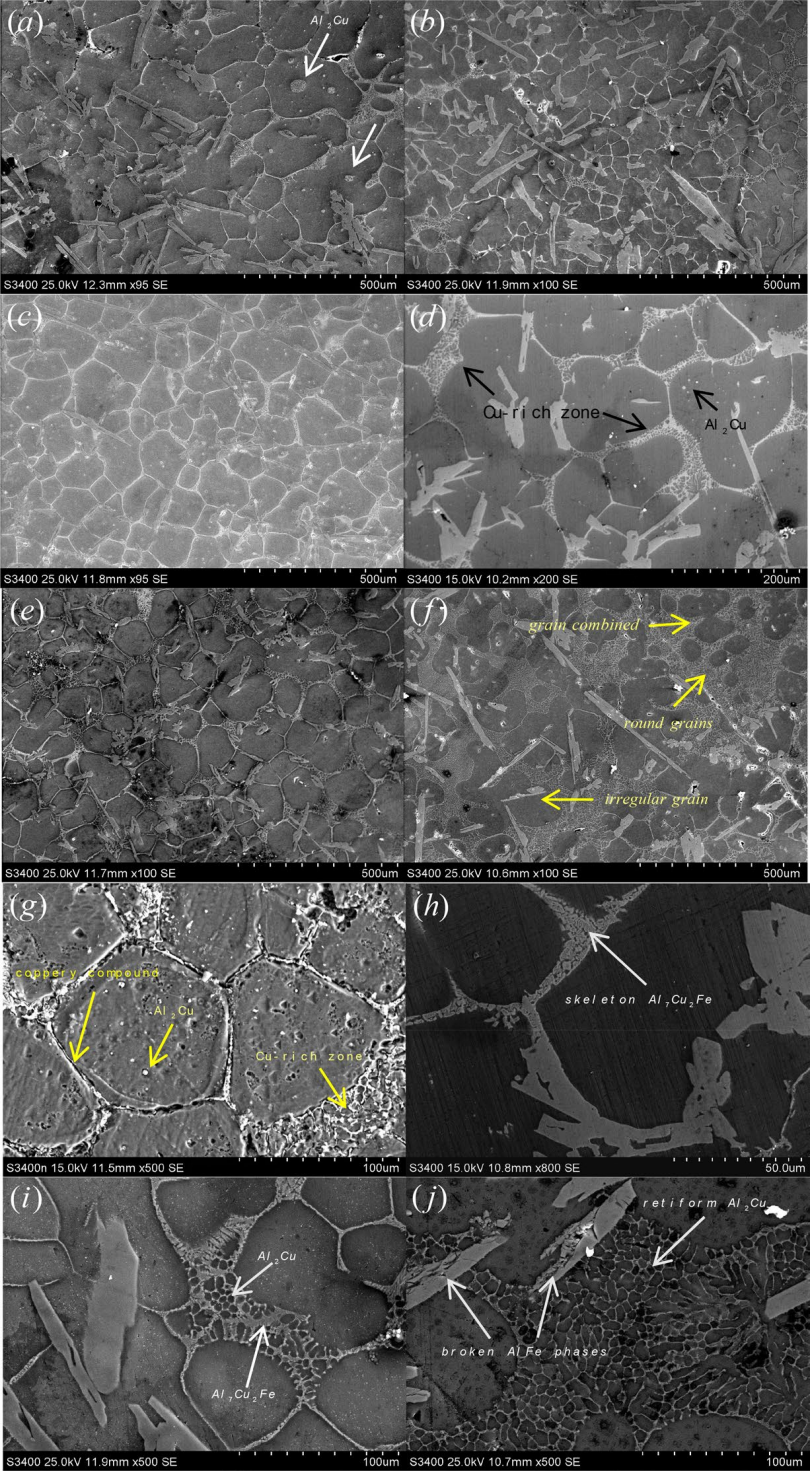
Microstructure of Al–Fe–Cu alloy semisolid heating under different temperatures and times (unetched). (**a**) Holding 30 min at 610 °C, there are many circular Al_2_Cu particles within the grains (from the liquid pools in the intra-grains) and thin the grain boundaries as well thick the re-solidified zones including Al_2_Cu and Al_7_Cu_2_Fe mixtures (Cu-rich zone); (**b**) holding 30 min at 630 °C; (**c**) holding 30 min at 640 °C, the rounded Al_2_Cu granules within the grains reduce and diminish, the grain boundaries broaden and the reticulations increase and the pinnates decrease in the re-solidified zones; (**d**) holding 5 min at 630 °C, there are retiform Cu-rich zone between the grains and rounded white Al_2_Cu particles in the intra-grains; (**e**) holding 15 min at 630 °C, the distribution of shorter and sparser AlFe phases results in more circular grains; (**f**) holding 60 min at 630 °C, upper right, the grains are completely surrounded by the liquid, forming rounded grains and occurring grains join. Lower left, the liquid is separated by AlFe phases, resulting in irregular grains; (**g**) holding 5 min at 640 °C (Keller etched), the white Al_2_Cu granules in the intra-grains, the white coppery compounds boundaries and retiform re-solidified zone between the grains; (**h**) holding 30 min at 610 °C, the skeleton Al_7_Cu_2_Fe etc. cupriferous compounds on the grain boundary; (**i**) holding for 20 min at 630 °C, the grain boundaries with skeleton cupriferous intermetallic and the re-solidified zone with the retiform Al_2_Cu and the penniform Al_7_Cu_2_Fe mixture; (**j**) holding 60 min at 640 °C, the retiform Al_2_Cu mixture on the re-solidified zone between the grains and AlFe phase, and showed broken AlFe phases.

**Figure 7:**
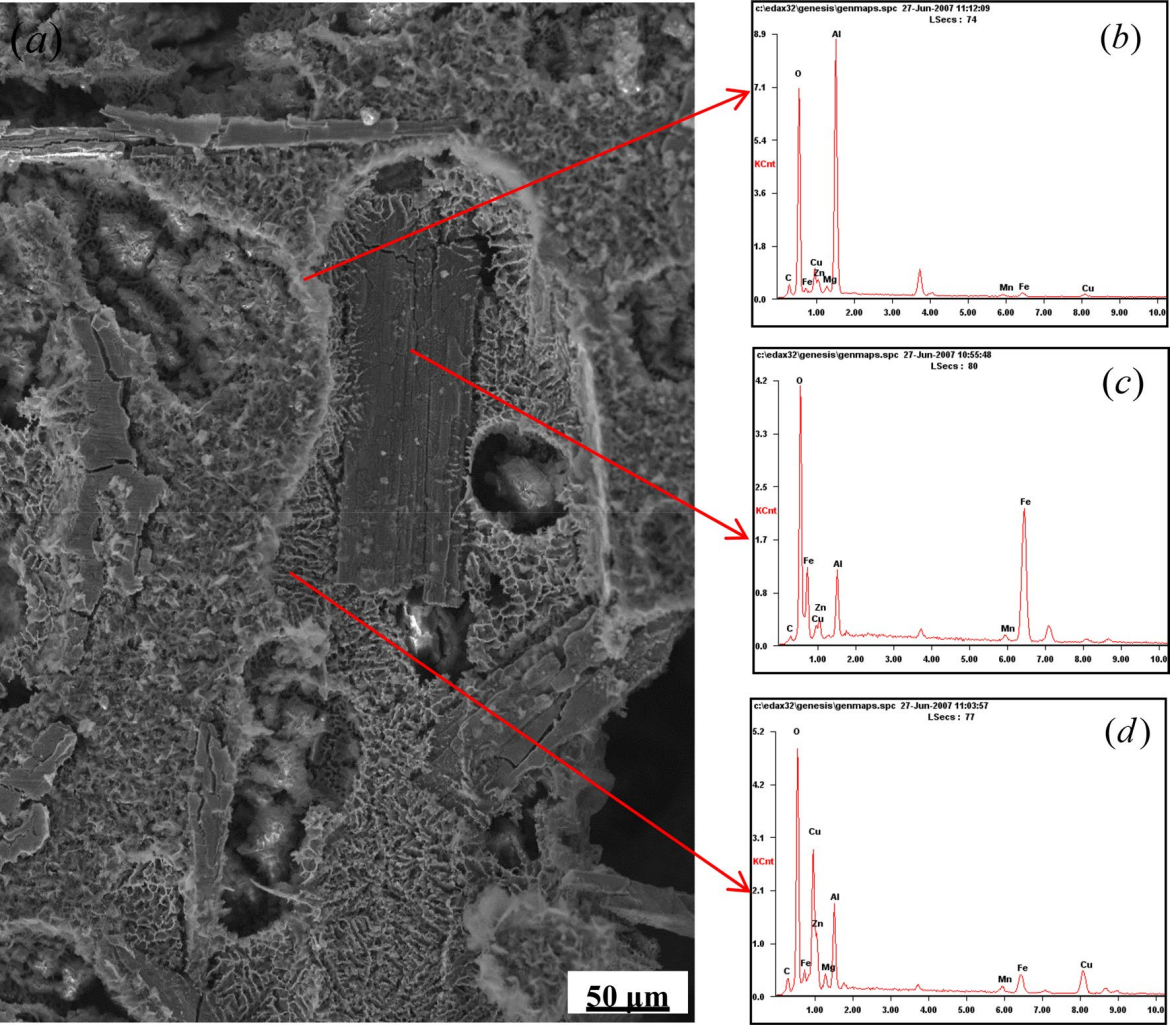
the quenched microstructure and EDS of Al–Fe–Cu alloy reheated at 630 °C for 40 min, deep etched. (**a**) Microstructure; (**b**) α-Al grain; (**c**) Fe-rich phase; (**d**) Cu-rich zone.

During the liquid–solid region heating, on the one hand, copper would diffuse from intra-grains to inter-grains or around AlFe phases and enrich in the liquid zones among the grains (second solidified or re-solidified zones). The diffusion of atomic species is closely associated with the heating temperature. For the samples held for 30 min at 610, 630, or 640 °C, the distribution of the major alloying elements varied in different ways (illustrated in Figs. 5a–c, 6a–c). It was observed that by increasing the heating temperature, a severe segregation of copper occurred at grain boundaries, in the liquid zones among the grains, and in the intra-granular liquid droplets (Fig. 5a). The number of Cu-rich phases decreases in the granular interior and increases in the grain boundaries or the second solidified zones because of the differential diffusion velocities for copper and aluminum atoms between the intra-grain and the intergranular regions. Figure 7 displays the microstructure of the Al–Fe–Cu alloy deep etched after heating at 630 °C for 40 min. As shown in Fig. 7, the α-Al grain corroded a cavity and white rich-Al edge, and the Cu-rich zones presented a fine mesh structure. The EDS analysis showed that there was more Cu among the grains in the as-reheated Al–Fe–Cu alloy than in the as-electromagnetic-stirred alloy. In most cases, the liquid among the grains could only migrate in a small range rather than from one zone to the other. Copper content in the re-solidified zones varied between 22 and 34 at%, indicating that AlFe phases significantly block the liquid migrating and causes copper segregation among the grains or around the Fe-rich phases. On the other hand, some undiffused copper was dissolved into the matrix with increasing holding time. When short-time or low-temperature heating, many white circular Al_2_Cu particles existed within the grains (Fig. 6a,d,e) because some eutectic liquid is easily entrapped within coarser α-Al grains during ripening^27^, and white reticular Cu-rich zones (re-solidified zones) appeared on the grain boundaries and around the AlFe phases (Fig. 6e). With increasing holding time, the cupreous compounds had transformed into continuous networks and close Chinese characters, and the Al_2_Cu particles could be barely caught within the grains (Fig. 6f). A previous study showed that when the Al–4Cu alloy was heated above 540 °C, the Cu dissolved in the matrix to form a stable supersaturated solid solution^28^. Distinctly, some Cu had re-dissolved into the matrix and the liquid during the liquid–solid region heating. The longer the holding time, the more Cu was enriched in the re-solidified zones or the grain boundaries. After reheating at 630 °C for 60 min, the Cu atoms have segregated into the re-solidified zones, and only a few Cu-rich particles were dispersed in the Al matrix. The content of Cu enriched in the liquid zone in inter-grain has nearly twice as many as that in intra-grain by employing EDS, which is consistent with previous findings^19,27^.

The re-solidified zones or the grain boundaries as shown in Fig. 6 could be typically described as reticular Cu-rich re-solidified zones that contain a higher liquid fraction, and the Cu-rich grain boundaries that feature a thin liquid film. The re-solidified zones gradually increased and the grain boundaries decreased with increasing holding temperatures and times. In other words, there were more alloying elements in the thicker re-solidified zones than those in the boundaries under the same holding temperature and time. For a specific isothermal holding condition, the retained solute in the melt formed at the lower temperature near the solidus was greater than that in the melt formed at the higher temperature upon reaching the expectations. On the other hand, the thickness of the grain boundaries significantly increased, as the temperature rose while holding at the same time. When more alloying elements were dissolved in the liquid rather than remaining in the solids, it exhausted the alloying elements in the adjacent solids. Therefore, the temperature of the solid was enhanced in these alloying element-depleted regions, that is, only by raising the temperature, the solid particles melt further. However, as the holding temperature increased, the segregation of copper also increased, which was because the diffusion velocities of the alloying elements in the alloy increased with increasing holding temperature. Moreover, the Cu-rich compounds in the grain boundary or the re-solidified zone are mainly skeletal or Chinese character-like Al_7_Cu_2_Fe at lower remelting temperatures according to EDS results (Fig. 6h). The Al_7_Cu_2_Fe compound decomposed or dissolved with increasing heating temperature or holding time resulting in the formation of a mixture structure consisting of reticular Al_2_Cu phases with Fe and Al_7_Cu_2_Fe intermetallic (Fig. 6i). The Al_7_Cu_2_Fe intermetallic in the mixtures markedly decreased with lengthening holding time or increasing the heating temperature (Fig. 6j), indicating that there is the reaction of the AlCuFe intermetallic decomposing into Al_2_Cu with Fe. It can also be seen in Fig. 5b–e that a few Fe segregations occur in the re-solidified zones with varying heating temperatures or times.

Furthermore, the solubility of both Cu and Fe in the Al–Fe–Cu alloy is interplayed by them, so that the solubility of both in Al is decreased severally. In addition, the Al_2_Cu eutectic temperature was reduced from 548.2 °C in the Al–Cu binary alloy to 537.5 °C in the Al–Fe–Cu alloy. During the liquid–solid region heating, low-melting-point phases such as Al_2_Cu and Al_7_Cu_2_Fe, was easily melted, making more Cu and Fe enrich in the liquid. Some Cu diffused to the grain boundaries and simultaneously in the intra-grains. The reticular re-solidified zones were formed at the early stages of heating in the liquid–solid region. This was also the main reason for the presence of thinner grain boundaries and thicker reticular re-solidified zones at a constant temperature. Owing to the variance of diffusion rates of both Al and Cu in Al at high temperatures, the Al atom preferred to concentrate in the convex parts of the grains, and the Cu atoms were discharged to the grain boundaries and enriched in the liquid zones. Most of the Cu-rich liquid assembled around the AlFe phases formed a structure where the Al_7_Cu_2_Fe phase was attached to the space around Al_13_Fe_4_ which is regarded as a nucleating core in ensuing solidification, the rest could solidify into rich Cu zones. This will hugely impact on the performance of the final product of Al–Fe–Cu semisolid formed alloy^22^.

In addition, it is worth mentioning that the segregations of Zinc synchronized with Copper, and the distribution was most in the re-solidified zones. As shown in Fig. 5, the increased level of manganese was synchronous with that of iron, indicating that manganese was abundant in the Fe-rich phase and was able to dissolve in Al_13_Fe_4_ or to form the Al_6_(FeMn) phase. According to the line scan analyses for magnesium in Fig. 5 and the elements scanning for magnesium, the magnesium atoms were uniformly distributed throughout the matrix, and this situation was hardly influenced by the liquid–solid region heating. Apparently, the effect of different semi-solid heating conditions on the distribution of magnesium is negligible.

### Coarsening kinetics

**Figure 8:**
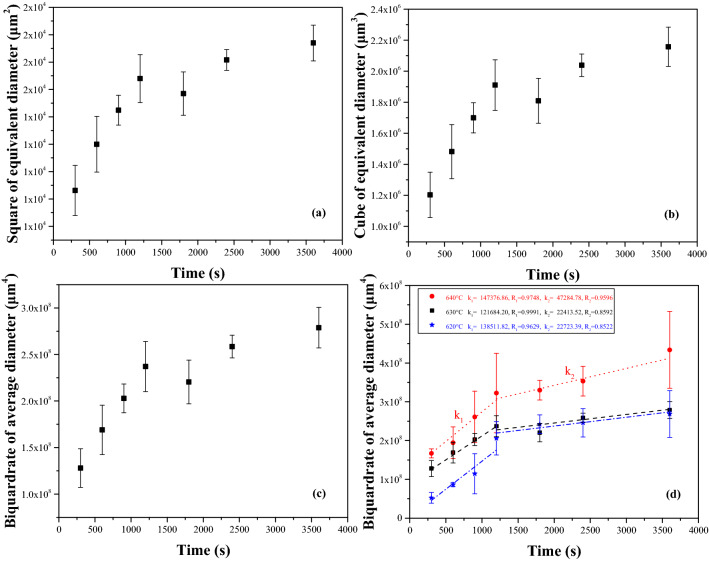
Plot of mean grain size (**a**) squared, (**b**) cubed and (**c**) quadruplicated as a function of holding time for Al–Fe–Cu alloy reheated at 630 °C, (**d**) relationship between quartic power of equivalent diameter and holding time for the alloy in various temperatures, lines represent the best linear fitted to the data. R is Pearson’s coefficient.

Coarsening data, for the Al–Fe–Cu alloy heating in liquid–solid region, of ***D***^***n***^ vs. ***t*** is plotted in Fig. 8 for various power exponents and heating temperatures. Pearson’s coefficients, ***R***, for best linear fits to the data, are given in Table 1, and ***R***s varied between 0.996 and 0.999 for the prime holding stage (0–20 min), and 0.851 to 0.859 for the later stage (20–60 min), respectively. The difference between the ***R*** values is small, and the corresponding higher values of ***R*** for various ***n*** values at different time buckets also differ. Although, the coarsening kinetics of semisolid alloys are still a controversial problem, the mean diameter (***D***) of the grains in various alloys after time ***t*** at the elevating temperature have confirmed the classical LSW equation, and the power exponent is approximately3 though varying between 2 and 4 during the liquid–solid region heating^5^. An increasing number of experts believe that the exponent of metallic coarsening in solid–liquid region is generally 3^5,7,10,13,18^, however, the best fitting is ***n*** = 4 according to the calculated results, coarsening was controlled by grain boundary diffusion.

**Table 1:** Variation of Pearson’s coefficients of the Al–Fe–Cu alloy heated at 630 °C with power exponents.

Soaking time (min)	Pearson’s coefficients (*R*)		
	*n* = 2	*n* = 3	*n* = 4
0–20	0.99601	0.99791	0.99911
20–60	0.85078	0.85476	0.85915

As shown in Fig. 8, two coarsening rate constants were obtained for ***n*** = 2, ***n*** = 3 and ***n*** = 4 at 630 °C or the soaking between 620 and 640 °C for ***n*** = 4. This indicated that there were the diverse growth rates in the different time buckets for Al–Fe-Cu alloy during heating in the liquid–solid region. The coarsening rate constants for the initial stage of the heating (5–20 min), ***K***_**1**_, and the later stage of the heating (20–60 min), ***K***_**2**_, were ***K***_**1**_ of 138,511.82 μm^4^/s and ***K***_**2**_ of 22,723.39 μm^4^/s at 620 °C, 121,684.20 μm^4^/s and 22,413.52 μm^4^/s at 630 °C, and 147,376.86 μm^4^/s and 47,284.78 μm^4^/s at 640 °C, respectively, were reckoned up according to data in Fig. 8d. The ***K***_**1**_ and the ***K***_**2**_ increased all with increasing holding temperature in the liquid–solid region, this result is in keeping with the findings in Ref. 7 and Ref. 15. The ***K*** is related to some material properties and responds to speed of the grain development or coarsening during heating. With heating temperature increasing, the liquid fraction in the alloy also increased and the diffusion quickened. The higher the liquid fraction, the more diffusion channels will be provided, and the diffusion in the liquid is faster than that in the solid^15^. The coarsening can be accelerated and the ***K*** value augment when a continuous liquid channel is formed around the grain. Moreover, the larger ***K***_**1**_ value in the initial soaking stage was mainly attributed to the liquid fraction increase with holding time at the temperature, because the liquid fraction of the Al–Fe–Cu alloy had verged on the equilibrium liquid fraction value at 630 °C (Fig. 11) after heating for 20 min (there was a liquid fraction of 27% at this time^2^). In addition, the variation of ***K***_**1**_ values were between 0.9 and 1.2 times with heating temperature increase, which is not very great, considering the alloy had a definite liquid fraction at a specific heating temperature. Furthermore, the smaller ***K***_**2**_ value of the later heating stage was ascribed to the mechanical blocking effect of AlFe phases on the liquid migrating and the solid turning. The ***K***_**2**_ value varied very little at lower temperature (620–630 °C) and more at higher temperature (640 °C), apparently, more the liquid would cancel out some of the blocking effect of AlFe phases at higher temperature. However, the ***K***_**2**_ value was several times less than the ***K***_**1**_ value for all testing temperatures, indicating that the obstruction of AlFe phases was obvious, it caused the grain growth to decelerate or even to cease in spite of the liquid tendency to saturate in the later stage of the heating.

Generally, remelting often starts at the interface of grain or phase boundaries during semi-solid heating. Due to the obstruction of AlFe phases, it was difficult for the adjacent grains to merge and develop further. Simultaneously, the nonuniform distributed liquid pools were not fully connected on soaking in the final period. As a result, the primary α-Al grains grew unevenly and the grains coarsened faster during the initial stage of soaking and then developed slowly. Furthermore, the grains surrounded by more liquid were rounding and those located in less liquid were irregular, and the grains in the alloy had different sizes and shapes. The grain growth was determined by AlFe phases, especially in the region of a few or few liquid films, and the grain coarsening rate decelerated in the later stage of heating.

In a word, the lamellate AlFe phases in the Al–Fe–Cu alloy block not only the grains growing and coarsening but also the liquid migrating from a liquid pool to another during heating in the solid–liquid region. The liquid was entrapped by the blocked AlFe phases, making the liquid around the α-Al grains distribute unevenly near the AlFe phases and do even far from the AlFe phases. The characteristic structure of the Al–Fe–Cu alloy led to that the circularity of the grains was inferior to the conventional semisolid Al alloys. In addition, the initial microstructure, the characteristic of grain boundaries and alloy compositions of Al–Fe–Cu alloy played a key role in grain coarsening in the solid–liquid region in addition to the mechanical inhibiting effect of AlFe phases^26^.

## Conclusions

During heating in the solid–liquid region, AlFe phases distributed randomly in the Al–Fe–Cu alloy deterred not only the α-Al grains further developing but also the liquid diffusing and merging. This makes, (1), the average diameter of grains increase gradually with increasing heating temperature or prolonging soaking time, and the smaller grains grow into the large grains but it has not developed further into the larger grains; (2), the liquid be difficult to interconnect, resulting in the liquid around the grains to distribute unevenly and the grains to spheroidize imperfectly, and the enrichment zone with various Cu contents form in the alloy; (3), the shape factor increase slowly and then decrease quickly with increasing the heating temperature or the holding time, both excessive temperature or overlong time degrade the ***SF***; (4), grains coarsening first increase quickly and following slowly with increasing holding time, coarsening rate constants in the later stage of heating (20–60 min) been a fraction of those in the initial stage of heating (5–20 min), coarsening controlled by a grain boundary diffusion. The grain sizes in the alloy presented a normal distribution.

Copper, zinc, iron, and manganese in addition to magnesium richen in the re-solidified zones among the grains and/or around the AlFe phases. Cu could diffuse from the intra-grains to the inter-grains and around AlFe phases besides redissolving in the matrix and dissolving in AlFe phases in Al–Fe–Cu alloy during the liquid–solid region heating. The higher the heating temperature or the longer the holding time, the more severe the segregation of Cu between the grains or around the AlFe phases, resulting in Cu between the grains outnumbering in the granular interiors. In addition, Zn in the alloy segregated simultaneously with Cu in the re-solidified zones, and Mn synchronized with Fe around AlFe phases to dissolve in Al_13_Fe_4_ or to form Al_6_(FeMn) phase. Mg was uniformly distributed throughout the matrix and this situation was scarcely affected by the semisolid remelting.

The optimum processing parameters for the Al–Fe–Cu alloy during reheating in the solid–liquid region are heating between 625 and 635 °C and holding 20–40 min, which is appropriate for thixoforming.

## Materials and methods

The nominal composition of experimental alloy is given in Table 2 and abbreviates Al–Fe–Cu alloy. It was melted by a 30 kW electrical resistance furnace controlling temperature automatically using a black-lead crucible and the smelting temperature range was from 750 to 900 °C. Pure Al (99.7%), Zn (99.9%) and Mg (99.9%) were used as raw materials. The industrially pure Al (99.7%), Fe (99.5%), Mn (99.7%) and Cu (99.95%) were used to fabricate the Al-20 wt% Fe, Al-10 wt%Mn and Al-50 wt% Cu master alloys. The trace elements Ti, Zr and B were added by the mixed salt consisted mainly of K_2_TiF_6_, KBF_4_, Na_2_ZrF_6_, KCl and NaCl analytical reagents. The chopped pure Al ingot, Al–Fe and Al–Mn master alloys were first melted in the electrical furnace. Other metal ingots Zn and Mg and the Al–Cu master alloy were added into the alloy melt at about 750 °C. The melt was covered by the mixed salt of approximately 1.4 wt% after melting. The melt was then heated up to approximately 850 °C and held for 20 min. The melt was stirring with a SiC rod for about 2 min and adjusting to approximately 770 °C. The dry broken C_2_Cl_6_ tablet (0.3 wt% of molten alloy) was degassing and standing about 10 min. After cleaning off the dross in the temperature range of 750–770 °C, the melt was poured into a graphite mold, which was located inside the home-made electromagnetic stirring apparatus and was pre-heated to 300 °C, and was electromagnetically stirred at different voltages before it completely solidified. The size of the cylindrical casting billet prepared by electromagnetic stirring is approximately Ф110 mm × 72 mm.

**Table 2:** Chemical composition of electromagnetic stirred billets for this study (wt%).

Composition	Fe	Cu	Zn	Mn	Mg	Zr	Ti	B	Al
Nominal	5.0–6.0	3.8–4.3	1.5–2.5	0.4–0.6	0.3–0.5	≤ 0.06	≤ 0.1	≤ 0.01	Bal
Average	5.41	4.0	1.90	0.46	0.41	0.055	0.010	0.0095	Bal
Standard deviation	0.417	0.119	0.215	0.063	0.043	0.0049	0.0064	0.00058	0.468

**Figure 9:**
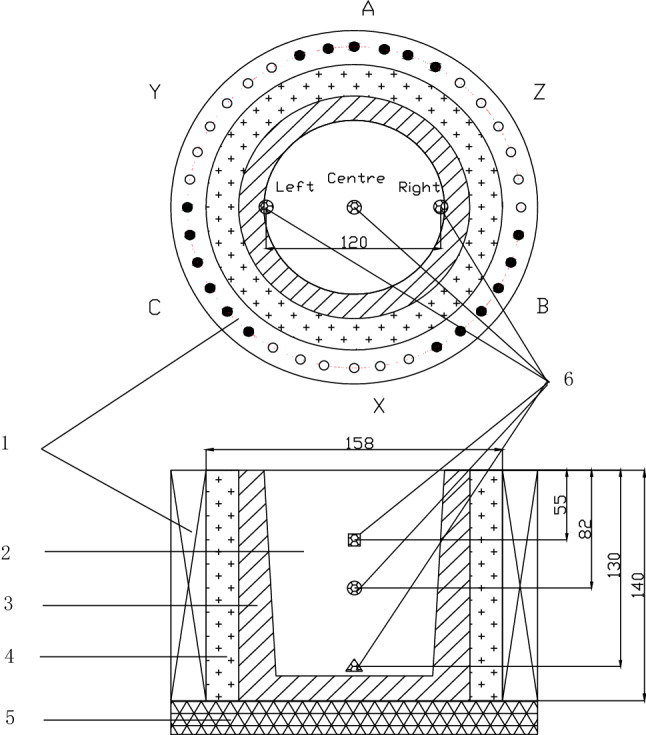
Schematic diagram of one of the home-made electromagnetic stirring apparatus^29^ (1) electromagnetic coils, (2) the melt; (3) graphite mold; (4) thermal insulation materials; (5) thermal insulation subplate; (6) measuring positions of the magnetic flux density.

**Figure 10:**
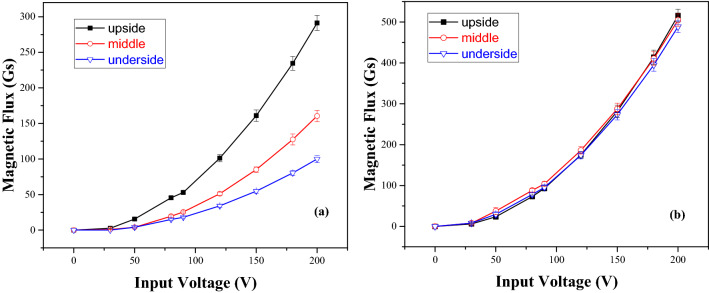
Measured magnetic flux of electromagnetic stirrer at idle load^29^ (**a**) centre location, (**b**) right location.

The home-made electromagnetic stirring apparatus made up of 36 electromagnetic coils and was controlled by using a three-phase AC voltage regulator, which the power of the stirrers is 9–15 kVA. There was a thermal insulation layer consisted of asbestos cloth and dry silica sand between the graphite mold ektexine and the inner wall of the stirrer (Fig. 9). The magnetic flux density (MFD) of the stirrer at idle load was measured by a hand-held Gaussmeter (resolution 0.1 Gs). It was found that the MFD increases nonlinearly with the input voltage and the MFD in the center of the stirrer was lower than that in the inner wall of the mold. The closer is to the inner wall of the stirrer, the higher the MFD is. Moreover, the MFD at the upside, at the middle and at the underside in the center of the stirrer was biggish difference, which the MFD at the upside was several times larger than that at the underside, but the MFD at those in the edges of the stirrer was basically same at the same voltage (Fig. 10). The MFD rose parabolically as the input voltage increased, i.e., the MFD was proportional to the square of the voltage. When the voltage 200 V, the MFD at the upside, at the middle and at the underside in the center of the stirrer was 291.2 Gs, 160.5 Gs and 100 Gs, respectively, which increased by 3–4 times compared with 70.1 Gs, 33.2 Gs and 23 Gs at the voltage 100 V, respectively. In addition, the MFD at the middle in the right edge and left edge of the stirrer was 471 Gs and 504.6 Gs, respectively, which the right edge is slightly higher than the left edge. The optimal stirring voltage is from 150 to 180 V for billets with diameter from 70 to 120 mm^29^.

The reheating specimens were cut from the casting billets, which it was prepared by electromagnetic stirring at voltage 180 V, and machined into the cylinders with diameter 10 mm and height 12 mm. The specimens were heated in a KOYO-52878 box-type resistance furnace at 200 °C/h to the scheduled temperature and then took 3 or 5 specimens and quenched them in cold water after holding the scheduled time until the experiment was completed at the temperature.

**Figure 11:**
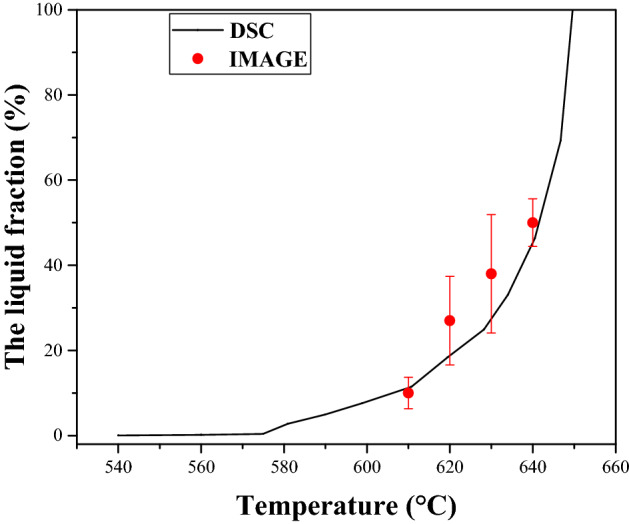
The liquid fractions of Al–Fe–Cu alloy by DSC and by Graphic observation for 30 min. (adapted from Ref.^2^).

The solid–liquid region of this alloy was measured by differential thermal analysis and was 505.2–649.9 °C. The DSC tests were carried out using a TA Q100 differential scanning calorimeter. DSC samples were pressed into smooth sheets with a diameter of 5 mm and a thickness of 1 mm, and put into a special copper crucible, and the samples were heated from room temperature to 900 °C at a rate of 10 °C/min and cooled to room temperature at the same rate. Meanwhile, a protective gas, pure nitrogen, was injected at a rate of 60 ml/min. Before the experiment, high-purity zinc (419.4 °C) and high-purity aluminum (660.37 °C) were used for melting point correction at the same rate. Figure 11 shows a plot of the isothermal holding temperatures and corresponding liquid volume fractions determined by differential scanning calorimetry (DSC) and the graphic method. Kim et al.^30^ believed that the release of the latent heat of solidification during solidifying is approximately proportional to the solidification amount of solid phase. Therefore, the area ratio of DSC exothermic curve can be used to determine the liquid–solid ratio of the alloy at different temperatures. The area integral of small step length was adopted to calculate the area ratio. The DSC heating rate was higher than the actual heating rate of specimens, which should rearward shift the endothermic peak in the curve, resulting in the phase transformations delaying and peak temperature increasing.

The liquid fraction, average grain size, and shape factor (***SF***) of the solid phase measured by means of an OLYMPUS BX60M optical microscope with ISA-4 image analyzer system. The equivalent (average) diameter and ***SF*** of solid grains are calculated in each state by applying the Eqs. (2) and (3)^31^.

2 $$ D = \frac{{\sum\limits_{i = 1}^{N} {\sqrt {\frac{{4A_{i} }}{\pi }} } }}{N} $$

3 $$ SF = \frac{{\sum\limits_{i = 1}^{N} {\frac{{4\pi A_{i} }}{{P_{i}^{2} }}} }}{N} $$

where ***D****, ****SF****, ****A****, ****N,*** and ***P*** are equivalent diameter, shape factor, area, quantity, and perimeter of solid particles, respectively. The grain size of each sample was measured by viewing ten different fields, each field had over five grains. The samples were etched by the Keller’s reagent, or deep corroded using 10% NaOH aqueous solution cooked at 80 °C for 10 min. The microstructure was observed by OLYMPUS BX60M optical microscope and HITACHI S3400n scanning electron microscope, which equipped with an energy dispersive X-ray spectrometer (EDX) to confirm the chemical composition of the phases. The phases were identified by using a Bruker D8 Discover X-ray diffractometer (XRD) using Cu Kα_1_ radiation.
